# Health Education and Promotion Interventions to Mitigate Geophagic Practise: A Scoping Review

**DOI:** 10.3389/phrs.2025.1607614

**Published:** 2025-04-30

**Authors:** Mohora Feida Malebatja, Moreoagae Bertha Randa, Mathildah Mpata Mokgatle, Oluwafemi Omoniyi Oguntibeju

**Affiliations:** ^1^ Department of Public Health, Environmental and Occupational Health Division, School of Healthcare Sciences, Sefako Makgatho Health Sciences University, Pretoria, South Africa; ^2^ Phytomedicine and Phytochemistry Group, Department of Biomedical Sciences, Faculty of Health and Wellness Sciences, Cape Peninsula University, Bellville, South Africa

**Keywords:** geophagia, geophagy interventions, women of reproductive age, health education and promotion, iron deficiency anaemia

## Abstract

**Objectives:**

This study aimed to review health education and promotion interventions that will assist in mitigating the practise of geophagy to reduce the potential health risks, mortalities and morbidities that are linked to geophagic practise.

**Methods:**

A scoping review was carried out guided by Arksey and O’Malley’s (2005) methodological framework and PRISMA reporting flowchart.

**Results:**

This scoping review found that health education and promotion interventions focusing on geophagy, nutrition, the uptake of iron and folic acid supplements, the potential health risks of geophagy, anaemia risk during pregnancy, oral and intravenous iron therapy and randomised trials are some of the methods that are currently used to prevent and control the practise of geophagy amongst women of reproductive age at antenatal care units, school and community level.

**Conclusion:**

It is concluded that the existing health education and promotion interventions that can assist women of reproductive age to cease the practise of geophagia are not effective. More emphasis should be given to the establishment of health education awareness programmes on the practise of geophagy, nutrition, and iron and folic acids uptake at community level.

## Introduction

Geophagia refers to the intentional ingestion of clay soil [1–5]. This is a common practise amongst women of reproductive age, men, the elderly, and children globally [6–8]. This is an ancient practise that is embedded in indigenous knowledge and culture [4, 6, 9, 10]. It is mainly considered as a socially acceptable practise that is motivated by different factors [9–12]. The physiological, nutritional, and health factors linked to the practise of geophagia are often not understood by consumers [9, 12, 13].

Geophagia is a serious major public health problem associated with maternal, neonatal, and childhood mortalities and morbidities [1, 14–16]. Geophagia is a behaviour that is known to lead to addiction over a prolonged time, which in turn can also lead to the dependency syndrome [13, 17, 18]. The potential health outcomes and dangers associated with geophagia include pregnancy complications, low birth weight babies, severe constipation, dental enamel, gastrointestinal infections, breathing complications, fatigue, worms’ infestation, malnutrition, lead poisoning, muscle weakness, bowel movement obstructions, oral mucositis, kidney damage, hypertension, appendicitis, leukemia, and iron deficiency [1, 3, 6, 8, 19–21].

Geophagia is mainly practised by pregnant women [1, 2, 22, 23]. It is not clear whether it is pregnancy that causes them to start consuming clay soil, or if clay soil ingestion is just one of the weird cravings that women often experience during pregnancy due to their bodies requiring increased intake of some nutrients, minerals and vitamins to cater for their unborn babies [15, 17, 24, 25]. It is also known that women of reproductive age who are not pregnant indulge in the practise of geophagia, but this must be for different reasons [2, 7, 9].

Various cultural, spiritual, environmental, physiological, social, economic and nutritional aspects contribute to the practise of geophagia [4, 9, 22, 26, 27]. In some countries and cultures geophagia is practised as part of their culture, tradition, and spirituality [2, 4, 5]. It is known to be a practise that is passed down from one generation to the next [6, 7]. A major contributing factor to this practise is the fact that consumers and the general public at large do not perceive geophagia as dangerous or that it is linked to potential health risks [4, 23].

Despite the availability of methods to curb the use of clay soil amongst women of reproductive age and the general population, it has still not been possible to completely persuade consumers to quit clay soil ingestion [1, 2, 28, 29]. It is imperative for the government, key role players, healthcare workers, traditional leaders and the public to holistically work together to mitigate the practise of geophagia [5, 15, 30]. The current public health interventions aiming at mitigating geophagia do not cover the environmental, health education and health promotion aspects [1, 7].

It is important to conduct this review to examine ways or approaches that could be adopted to effectively prevent and control the possible negative health outcomes associated with geophagia such as maternal and child mortalities, which are listed amongst the sustainable development goals of 2019 targeted at reducing maternal and childhood mortalities [31]. Therefore, the principal aim of this review is to explore health education and promotion interventions that could be used to mitigate the practise of geophagia.

## Methods

### Study Design

This scoping review followed Arksey and O’Malley’s framework and the Joanna Briggs Institute scoping review methodology [32]. The review process comprised the stages outlined in the table below (Table 1). This design of the review was adapted from Arksey and O’Malley’s framework, incorporated with PRISMA-ScR [33].

**TABLE 1:** Stages of Arksey and O’Malley’s framework and Preferred Reporting Items for Systematic reviews and Meta-Analyses extension for Scoping Reviews (Worldwide, 2024).

Arksey and O’Malley’s framework	PRISMA-ScR protocol relevant checklist items
Identify the research questions	Objectives
Identify the relevant studies	Information sources, eligibility criteria, and search strategy
Selection of studies	Selection of sources for evidence purpose
Data extraction	Process of data charting, data items, and critical appraisal
Summarising the findings	Summarising the results

### Identifying the Research Question

A preliminary exploratory search of the literature was conducted to develop the research questions. The primary search focused on identifying health education and promotion interventions aimed at mitigating the practise of geophagia. Following the primary search, various research objectives were developed as stated below to identify the interventions aimed at mitigating geophagia amongst women of reproductive age, to describe the available health education and promotion initiatives for geophagia, too explore any possible relationship between the practise of geophagia and the development of health outcomes, to identify the potential health risks associated with the practise of geophagia amongst women of reproductive age, and to describe the available methods used to prevent, treat and control the practise of geophagia. The research question for this scoping review was phrased as “What are the health education and promotion interventions used to mitigate the practise of geophagia”.

### Identifying Relevant Studies

The review was conducted using both published peer reviewed journal articles and grey literature following the Prisma reporting template and the Arksey and O’Malley framework. [32, 34]. A systematic search of studies published between 2020 and 2024 on health education and promotion interventions to curb the practise of geophagia was utilised. The online databases that were consulted included Scopus, PubMed, Science Direct, and Google Scholar. Related articles from the literature retrieved were consulted to identify more articles. The following key words; geophagia, health education and promotion, women of reproductive age, geophagia interventions, iron deficiency anemia, and interventions were used in the search strategy. The review included studies conducted in the last 5 years to give a balanced view of the field, backed up by evidence. An intense literature review search was performed using Scopus, Science Direct, Google Scholar, and the University Repository website.

### Search Strategy

Table 2 outlines a comprehensive search strategy for this review. The principal researcher together with the research team, supervisors, and librarian assisted in determining the search terms. Keywords such as geophagia interventions, health education and promotion, geophagia, women of reproductive age, iron deficiency anaemia, and interventions were used to search for literature published from 2020 to 2024 using electronic databases such as Science Direct, Google Scholar, pubMed and Scopus. Advanced search and normal search strategies were employed using combinations of keywords to increase the chances of retrieving more literature. The articles retrieved via this search were screened according to titles, abstracts, and intervention types. Endnote Reference Manager software was used to store and import the articles for tracking, record keeping and the creation of a list of references.

**TABLE 2:** Selection criteria for inclusion and exclusion (Worldwide, 2024).

S. No.	Category	Inclusion criteria	Exclusion criteria
1	Language	English	Other language
2	Publication duration range	2020 to 2024	Before 2020
3	Geographic location	All countries	
4	Population category	Geophagic women of reproductive age	Geophagic men, children, and the elderly
5	Study title	Studies on geophagia, health education and promotion interventions	Studies not addressing geophagia health education and promotion interventions
6	Study designs	All studies that used study designs such as cohort, case control, cross-sectional, intervention studies, quasi experimental, randomised controlled trials, qualitative and quantitative	Review studies (meta-analyses, systematic reviews, and scoping reviews)

#### Setting

This review focused on geophagia health education and promotion intervention studies conducted worldwide.

#### Study Selection

**FIGURE 1:**
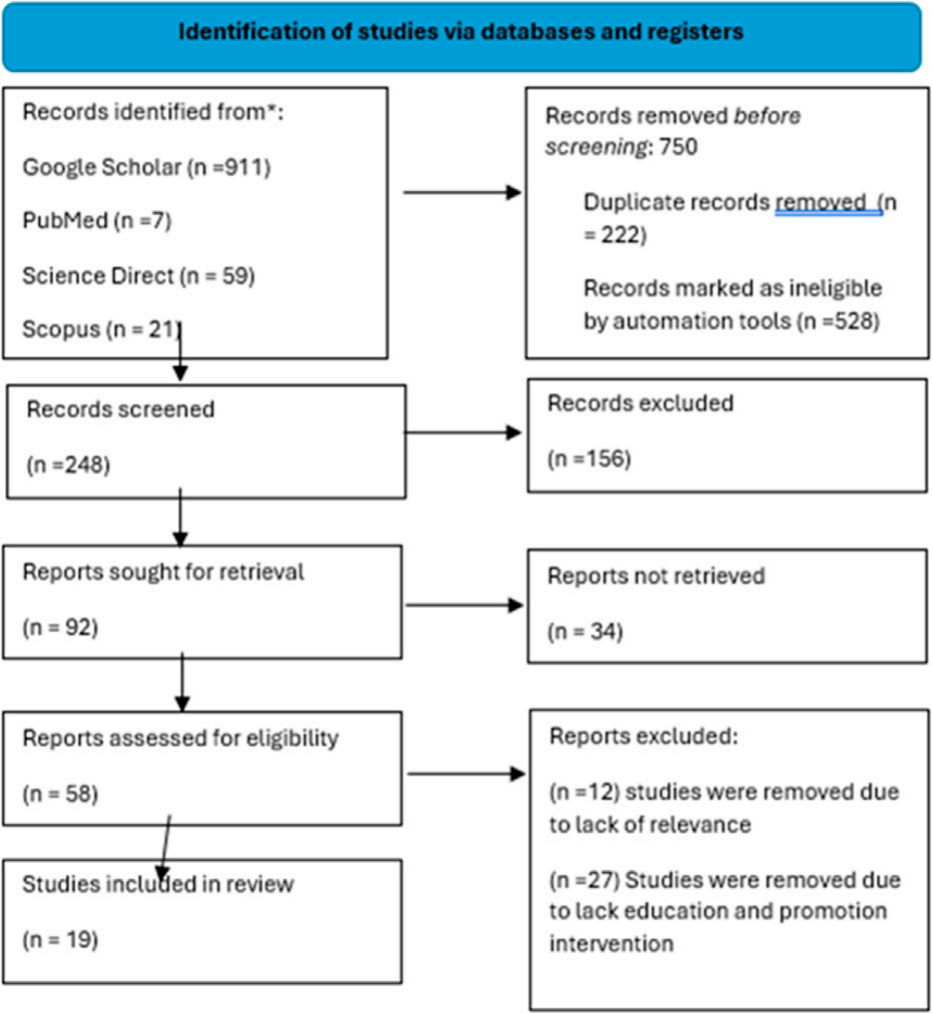
Flow diagram of search for, screening of and selection of published research studies (Globally, 2024).

The researcher and the research team separately reviewed journal articles according to the titles, abstracts and intervention type. They all scrutinised the studies to ensure their compliance with the set criteria for inclusion. Studies that qualified to be included in this review were those that assessed geophagia health education and promotion interventions. These studies were published in English with full in text open access. The research team held meetings to discuss potential discrepancies during the review in order to reach agreement on the interpretation of the criteria for selection. The screening of journal articles took place in two stages. In the first stage the reviewers separately assessed the titles and abstracts of all the retrieved citations against set inclusion criteria outlined in table 2. Where articles were deemed pertinent by one or more of the reviewers, they were presented to all the reviewers in order that agreement could be reached for those articles to be included in the full text review. Articles were also screened to allow the removal of duplicates from different databases according to their titles and abstracts. Duplicate articles were deleted, and the remaining articles that did not meet the set criteria were also excluded. The articles that satisfied the eligibility criteria for inclusion were reviewed in full (See Table 3). In case the reviewers differed, a resolution was taken through discussion or third-party consultation where necessary. The Preferred Reporting Items for Systematic Reviews and Meta-Analyses (PRISMA) flowchart template was used to perform the study selection process. See Figure 1.

**TABLE 3:** Relevant studies from Google Scholar, Public Medline, Science Direct, and Scopus that were included in this scoping review (Globally, 2024).

Authors	Intervention types	Study design	Location and year
Lavanya P, et.al	Adherence to iron and folic acid supplementation among antenatal mothers attending a tertiary care center, Puducherry	Mixed methods study	Puducherry India 2020
Njiru, H., et al.	Effectiveness of public health education on the uptake of iron and folic acid supplements among pregnant women: a stepped wedge cluster randomised trial	Cluster randomised trial	Embu County, Kenya
Abdisa, D.K., et al.	Effect of community based nutritional education on knowledge, attitude and compliance to IFA supplementation among pregnant women in rural areas of southwest Ethiopia: a quasi-experimental study	Quasi experimental study	Ethiopia 2023
Gebremichael, T.G. and T.G. Welesamuel	Adherence to iron-folic acid supplement and associated factors among antenatal care attending pregnant mothers in governmental health institutions of Adwa town, Tigray, Ethiopia: Cross-sectional study	Cross-sectional study	Ethiopia 2020
Singh, P.K., et al.	Public health interventions to improve maternal nutrition during pregnancy: a nationally representative study of iron and folic acid consumption and food supplements in India	Nationally representative study	India 2020
Mogongoa, L	Efficacy of oral iron therapy in geophagic women with iron deficiency anaemia residing in Botshabelo, South Africa	Prospective randomised intervention study	Botshabelo, South Africa 2020
Mogongoa, L. F	Comparison of oral and intravenous iron therapy in geophagic Botshabelo women with iron deficiency anaemia	Prospective randomised intervention study	Botshabelo, South Africa 2020
Sanghvi, T.G., et al.	Comprehensive approach for improving adherence to prenatal iron and folic acid supplements based on intervention studies in Bangladesh, Burkina Faso, Ethiopia, and India	Mixed methods study	Bangladesh, Burkina Faso, Ethiopia, and India 2023
Huda, T.M., et al.	Effect of early use of maternal iron and folic acid supplements on neonatal survival: a community-based cluster randomised controlled trial in rural Bangladesh (Shonjibon Trial)	Community-based cluster randomised controlled trial	Rural Bangladesh 2022
Kamau, M.W., et al.	Effect of a community-based approach of iron and folic acid supplementation on compliance by pregnant women in Kiambu County, Kenya: a quasi-experimental study	Quasi-experimental study	Kiambu County, Kenya 2020
Yamashita, T., et al.	Maternal knowledge associated with the prevalence of iron and folic acid supplementation among pregnant women in Muntinlupa, Philippines: a cross-sectional study	Cross-sectional study	Muntinlupa, Philippines 2021
Seminar, A.U., et al.	Awareness about anaemia and Weekly Iron-Folic Acid Supplementation (WIFAS) among school-going adolescent girls and parents in East Java and East Nusa Tenggara, Indonesia	Qualitative study	East Java and East Nusa Tenggara, Indonesia 2020
Gosdin, L., et al.	A school-based weekly iron and folic acid supplementation program effectively reduces anaemia in a prospective cohort of Ghanaian adolescent girls	Prospective cohort	Ghana 2021
Hasan, M.M., et al.	Anaemia in women of reproductive age in low- and middle-income countries: progress towards the 2025 global nutrition target.	Secondary analysis, using repeated cross-sectional surveys	Low- and middle-income countries 2022
Haile, B., et al.	Factors associated with compliance with weekly iron and folic acid supplementation among school adolescent girls in Debub Achefer district, northwest Ethiopia: School-based cross-sectional study	School-based cross-sectional study	Northwest Ethiopia 2024
Ansari, M.R., et al.	The acceptability of weekly iron-folic acid supplementation and its influencing factors among adolescent school girls in Yogyakarta city: a mixed methods study	Mixed methods study	Yogyakarta City, Indonesia 2021
Bali, S. and Y. Alok	Is ignorance of the weekly iron and folic acid scheme among adolescents the deciding factor for its suboptimal utilization and ineffectiveness? A cross-sectional study	Cross-sectional study	Madhya Pradesh, India 2022
Biswas, B., et al.	Barriers, facilitators of iron and folic acid supplementation, and deworming program among school-going adolescents of Deoghar, Jharkhand, India: a mixed-methods study	A mixed methods study	Deoghar, Jharkhand, India 2024
Wangaskar, S.A., et al.	Prevalence of anaemia and compliance to weekly iron-folic acid supplementation programme amongst adolescents in selected schools of urban Puducherry, India	A cross-sectional study	Urban Puducherry, India 2021

#### Data Extraction

A data extraction framework was developed to guide the eligible full texts retrieved in the literature review, based on the initial scoping phase. Since the researcher and research team served as reviewers, each one used a standardised data extraction form separately to draw from the articles that were considered for inclusion. A table was drawn to include information on geophagia health education and promotion interventions to mitigate the practise of geophagia, the methodology used, the sample size and population group of interest. The important information that was covered by the form included the design of the study, the year of publication, the title, the geophagia health education and promotion interventions investigated, the country where the study was conducted, and the main findings. For consistency, all reviewers scheduled a pilot test of the studies included, and the categories that needed to be amended or merged. Where reviewers differ in their opinions, an intervention discussion amongst the team was convened to reach an agreement.

#### Summarising the Findings

A narrative synthesis was used to summarise the existing literature on geophagia health education and promotion interventions and to propose the most effective strategies to curb and mitigate the practise in order to reduce the incidence of maternal, neonatal and childhood mortalities and morbidities. This review provided a thematic overview of the findings of the studies that have investigated geophagia health education and promotion interventions. The studies were summarised, interpreted and reported, using a standardised tool, to add to the body of relevant knowledge. In addition, gaps in the existing literature were identified in order to be able to make pertinent suggestions about future research topics. The findings of this review could be used to inform policy formulation and strategy development for campaigns aimed at persuading people to quit engaging in geophagia. Furthermore, the findings of this study could be used to inform the development of health education and promotion awareness programmes aimed at mitigating the effects of geophagia.

#### Bias

To minimise risk of bias, this review used PRISMA reporting template to select and include studies in this review to minimise risk to bias. This is a flow diagram checklist is used to minimise risk of bias, since it outlines on screening processes followed, how studies were searched, screened, included and reasons for inclusions. This tool offers benefits such as transparency, and replication.

#### Ethics Approval of the Research Project

Since this review was based on publicly available publications and materials, no ethical approval was required.

## Results

### Significance of Study and Dissemination

The findings of the review will be distributed to health ministries and policymakers in order to inform policy direction on the health outcomes of geophagia. The findings will also be disseminated to the general public through the use of community engagements and awareness campaigns. The outcome of the review was a summary of the existing geophagia health education and promotion interventions.

## Discussion

Generally, there are no adequate health education and promotion interventions that are aimed at mitigating geophagy amongst women of reproductive age worldwide [15, 35, 36]. Presently, there are different methods and initiatives used to curb iron deficiency anaemia, which is known to be the leading cause of geophagic practise amongst women of reproductive age worldwide [3, 24, 35, 37]. Epidemiological evidence suggests the existence of a causal relationship between the development of iron deficiency anaemia and the practise of geophagy [38, 39]. Studies reveal that the interventions and medications administered to iron-deficiency anaemia patients are similar to those given for the prevention, control and management of geophagy amongst the general population [29, 36, 40–42].

### Health Effects of Geophagic Practise

Studies have shown that the practise of geophagy is linked to various health conditions such as appendicitis, cancer, hypokalaemia, fatigue, shortness of breath, dental damage, perforation of sigmoid colon, constipation, hypertension, lead poisoning, gastrointestinal infections, oral mucositis, bowel movement obstructions, skin sores, muscle weakness, appendicitis, leukemia, kidney damage, electrolyte imbalance, malnutrition, hepatic and renal damage, and hookworm infection [1, 3, 4, 20, 22, 43–46]. Further complications related to the practise of geophagy include iron deficiency anaemia, pregnancy complications, pre-mature birth, dysfunctional labour, miscarriages, stillbirth, low birth weights, and pre-contamination of the foetus [1, 5, 22, 43, 47, 48]. The practise of geophagy gives rise to teratogenic risks that could result in birth defects leading to increased child morbidity and mortality [4, 5, 20, 23, 48, 49].

### Health Education Interventions

#### Public Health Education on the Uptake of Iron and Folic Acid Supplements at Antenatal Care

Studies reveal that public health education on the uptake of iron and folic acid supplements during pregnancy has effectively reduced the practise of geophagy amongst women of reproductive age [50, 51]. Women of reproductive age who attend public health education on the uptake of iron and folic acid supplements are more likely to make healthy pregnancy choices than those who lack proper knowledge and understanding of the iron and folic acid supplements [50–52]. Public health education on the uptake of iron and folic acid supplements should be considered on different platforms to lessen the practise of geophagy [16, 51].

#### Community-Based Health Education on Knowledge of and Attitudes Towards Iron and Folic Acid Supplementation Amongst Pregnant People

A study recommends a community-based health education programme on knowledge and attitudes towards iron and folic acid supplementation amongst pregnant women as an intervention strategy that could be employed to curb the practise of geophagy amongst pregnant women [53]. The offering of nutrition education as part of the community-based health education on knowledge and attitudes towards iron and folic acid supplementation has been reported to be effective in mitigating the practise of geophagy amongst pregnant women [29, 36, 51].

#### Community-Based Nutrition Health Education Targeting Geophagy

Community nutrition education targeting geophagy is one of the initiatives that could be used to mitigate the practise of geophagy [54]. It is reported that many geophagic women lack proper knowledge and understanding of their physiological and nutritional needs, particularly their needs when pregnant [27–29, 36]. Pregnant women are taught about the correct food choices, plans and diets that will promote their pregnancy outcomes during their consultation in the antenatal care service units, but the topic of geophagy has never raised [15, 41, 51]. The delivery of geophagy health education by healthcare workers and dieticians to geophagic women of reproductive age is pivotal to preventing iron deficiency anaemia, which is usually linked to the practise of geophagy [15, 36, 55, 56].

#### Health Education About Anaemia Risks and Geophagy to Promote Good Pregnancy Outcomes at Antenatal Care

Another intervention method that could be used to prevent and control geophagy would be the implementation of health education about anaemia risks and geophagy to promote pregnancy outcomes at antenatal care units for pregnant women as the major consumers of clay soil [15, 51, 55, 56]. Educating pregnant women at the antenatal care units could minimise the risk of them developing iron deficiency anaemia and could reduce the practise of geophagy amongst pregnant women and the public at large [4, 7, 18, 51].

#### Geophagy to Form Part of a Nutrition Health Education Package at Antenatal Care

Studies have indicated that educating women about the harmful effects of geophagy at the antenatal care units can be employed as one of the health education intervention programmes aimed at preventing, controlling, and treating the practise of geophagy amongst pregnant women [1, 18, 27, 57]. The concept of geophagy would be explored and taught to pregnant women at a deeper level to capacitate these women to take informed decisions when it comes to the practise of geophagy [7, 17, 23, 58]. The incorporation of geophagy in the nutrition health education package would assist in decreasing the practise amongst pregnant women [1, 2, 23, 57]. A study emphasises the need for healthcare workers to intensify health education on geophagy and its associated negative health effects on pregnant women and the foetus in all trimesters of gestation [2].

#### Potential Health Risks Education on Chemicals Found in Clay

Studies recommend the provision of education on the potential health risks associated with the chemicals found in clay, to educate geophagic women about the dangerous health-related risks and outcomes resulting from the toxicological aspects of the practise of geophagy [5, 17, 23, 43, 59]. Potential health risks education on the chemicals found in clay soil at community, school, and public healthcare level offers a crucial opportunity to decrease the practise of geophagy [4, 59].

### Health Promotion Interventions

#### Oral and Intravenous Iron Therapy

The health promotion interventions aimed at preventing, controlling, and treating the practise of geophagy include the administration of oral iron and intravenous therapy [42]. Oral iron therapy is often administered to pregnant women at the antenatal care units and sold over the counter from pharmacies to the general public [14, 25, 36, 42]. Other studies report that many pregnant women with a history of practicing geophagy are often faced with challenge of iron deficiency anaemia, which necessitates the need for intravenous iron therapy post childbirth [14, 27, 35, 49].

#### Replacement of Geophagic Material With Food Items

Studies have shown that the replacement of clay soil with food items could aid in reducing the practise of geophagy [28, 36]. Adolescent women were encouraged to eat any snack or food item whenever they experienced the urge to ingest clay soil [28, 60, 61]. This intervention has yielded positive results, proving to be effective in decreasing the practise of geophagy amongst women of reproductive age [13, 28, 36]. Most of the adolescent women who were participants in this intervention programme reported that they had managed to quit geophagy [28, 60, 61]. Additionally, it was reported that the desire to practise geophagy had literally disappeared after replacing the clay soil with a food item each time they felt the craving [28, 36].

#### Folic Acid and Iron Supplements Implementation at Antenatal Care Centres for Pregnant Women

Offering pregnant women folic acid and iron supplements is one of the health interventions that is aimed at promoting maternal and child health [31, 41]. Anaemia amongst women of reproductive age is of public health importance [60, 61]. Preventative and control measures of anaemia amongst women of reproductive age such as the provision of folic acid and iron supplements at antenatal care are presently among the methods used to minimise the practise of geophagy [14–16, 31, 42], however, some women do not consume folic acid and iron supplements after collection in from the antenatal care units [16, 35, 50, 62].

#### Folic Acid and Iron Supplementation Integration in High School Health Education and Nutrition Programmes

There are nutrition and health education programmes that are presently being implemented at school level to facilitate awareness amongst pupils [12, 60, 61, 63]. The addition of folic acid and iron supplementation as part of the health education programme has been identified as one of the key initiatives that could be considered to promote the mitigation of the practise of geophagy amongst women of reproductive age [60, 63–65]. This intervention programme targets adolescent women at secondary schools, where health education about anaemia, menstruation, iron nutrition and geophagy are covered as among the main topics that need to be addressed [60, 66, 67]. This intervention has generated positive results in terms of adolescent women quitting the practise of geophagy [12, 61].

#### Environmental Health Interventions

Other studies advocate the removal and pre-treatment of toxic elements found in clay soil that is eaten by geophagic women of reproductive age to prevent possible detrimental health complications [1, 5, 59]. The removal of the toxic elements found in clay soil before its sale would not contribute to the decline of geophagy amongst women of reproductive age, but it would reduce the level of toxicity, which often leads to serious health problems [1, 5, 17, 59]. Another study recommends the biomonitoring of clay soil consumers, particularly of women of reproductive age, to confirm the suspected health effects of the geophagic practise [5].

### Other Interventions

#### Further Research and Multidisciplinary Research

Studies have stressed the need for more research pertaining to the practise of geophagy to fully understand the etiology, physiological, nutritional, psychological, medical and spiritual impact of the practise amongst women of reproductive age, with an emphasis on pregnant women as the identified vulnerable group [7, 20, 30]. A multi-disciplinary research project would bring together different disciplines to address the practise of geophagy as a societal problem [7, 20]. If more multidisciplinary research were to be conducted pertaining to the practise of geophagy, the public would have more access to evidence-based information that could be used to inform their decisions when it comes to the practise of geophagy.

### Conclusion

It is concluded that the existing health education and promotion interventions aimed at mitigating the practise of geophagia amongst women of reproductive age, such as administration of iron and folic acid supplementation are effective. More emphasis must be given to the establishment of health education programmes dealing with the practise of geophagia, nutrition, and iron and folic acids uptake at community level, schools, churches, antenatal care services, and primary healthcare centres to discourage the practise of geophagy amongst women of reproductive age. Another conclusion can be drawn, that multi-disciplinary research must be prioritised at research institutes and academic institutions to address the practise of geophagy as a societal problem.
